# Clinoenstatite/Tantalum Coating for Enhancement of Biocompatibility and Corrosion Protection of Mg Alloy

**DOI:** 10.3390/jfb11020026

**Published:** 2020-04-13

**Authors:** Hamid Reza Bakhsheshi-Rad, Aliakbar Najafinezhad, Esah Hamzah, Ahmad Fauzi Ismail, Filippo Berto, Xiongbiao Chen

**Affiliations:** 1 Advanced Materials Research Center, Department of Materials Engineering, Najafabad Branch, Islamic Azad University, Najafabad, Iran; aliakbar.najafinejad@gmail.com; 2 Department of Materials, Manufacturing and Industrial Engineering, Faculty of Mechanical Engineering, Universiti Teknologi Malaysia, Skudai 81310, Johor Bahru, Johor, Malaysia; esah@mail.fkm.utm.my; 3 Advanced Membrane Technology Research Center (AMTEC), Universiti Teknologi Malaysia, Skudai 81310, Johor Bahru, Johor, Malaysia; afauzi@utm.my; 4 Department of Mechanical and Industrial Engineering, Norwegian University of Science and Technology, 7491 Trondheim, Norway; 5 Department of Mechanical Engineering, College of Engineering, University of Saskatchewan, Saskatoon, SK S7N 5A9, Canada; xbc719@mail.usask.ca

**Keywords:** tantalum nitride, clinoenstatite, sputtered films, EPD, biocompatibility, corrosion protection

## Abstract

Biodegradable Mg alloys have appeared as the most appealing metals for biomedical applications, particularly as temporary bone implants. However, issues regarding high corrosion rate and biocompatibility restrict their application. Hence, in the present work, nanostructured clinoenstatite (CLT, MgSiO_3_)/tantalum nitride (TaN) was deposited on the Mg-Ca-Zn alloy via electrophoretic deposition (EPD) along with physical vapor deposition (PVD) to improve the corrosion and biological characteristics of the Mg-Ca-Zn alloy. The TaN intermediate layer with bubble like morphology possessed a compact and homogenous structure with a thickness of about 950 nm while the thick CLT over-layer (~15 μm) displayed a less compact structure containing nano-porosities as well as nanoparticles with spherical morphology. The electrochemical tests demonstrated that the as prepared CLT/TaN film is able to substantially increase the anticorrosion property of Mg-Ca-Zn bare alloy. Cytocompatibility outcomes indicated that formation of CLT and TaN on the Mg bare alloy surface enhanced cell viability, proliferation and growth, implying excellent biocompatibility. Taken together, the CLT/TaN coating exhibits appropriate characteristic including anticorrosion property and biocompatibility in order to employ in biomedical files.

## 1. Introduction

Recently, magnesium based-alloys have received a great deal of consideration as potential biodegradable materials for implants because of their excellent mechanical characteristics and biodegradability performance [1,2,3]. It is noted that the Mg containing Ca and Zn has the necessary criteria to be employed as an orthopedic biodegradable material and Zn and Ca are capable of improving the healing rate of broken or impaired bones by creating hydroxyapatite throughout the degradation of the alloy in the system [4,5,6,7]. Having said that, the high degradation rate of Mg alloys in physiological medium lessens the mechanical characteristics of implants ahead of time and generates Mg (OH)_2_ and H_2_ gas [8,9]. An additional issue is whether or not the Mg alloy is able to maintain mechanical characteristic and biocompatibility throughout the implantation period [10,11].

Lately, numerous coating approaches [12,13,14,15] including PVD and EPD methods have already been applied to escalate the anticorrosion property and cytocompatibility of the Mg-based alloy. In present work, a two-step process was performed for the formation of CLT and TaN on the Mg bare alloys surface. Initially, PVD, a thoroughly clean and eco-friendly approach, was appointed to create TaN as an under- layer with a dense structure to enhance corrosion resistance [16,17].

In this context, porous Ta-based materials has been employed in orthopedic applications (total hip replacement) as a result of formation of a uniform, dense and cytocompatible of Ta_2_O_5_ film on their surface [18,19]. Tantalum presents great inertness in numerous aggressive media because of the creation of uniform, stable and dense Ta_2_O_5_ layer on the substrate surface. Like some other transition metals, including Ti, and Zr, tantalum presents a great affinity for nitrogen, which may react with tantalum to create inert TaN and enhance the anticorrosion properties.

Subsequently, EPD as a highly flexible, effortless and the most affordable approach was performed to fabricate clinoenstatite (CLT; MgSiO_3_) as the top-layer to enhance formation of apatite and biocompatibility of the Mg alloy substrate [20,21]. CLT is part of Mg containing silicate-based ceramic groups and is applied for artificial bone and dental root because of its outstanding formation of calcium-phosphate, biocompatibility and better mechanical characteristics such as adhesive strength and fracture toughness in comparison with hydroxyapatite [22,23]. However, the presence of microcracks and micropores in the outer layer owing to the CLT film is typically porous, which may offer pathways for penetration of the SBF into the coating throughout corrosion process which reduce corrosion resistance of substrate. To solve this issue, TaN as inner-layer with a compacted structure has the ability to protect the substrate when the SBF pass through from the pore structure of the out-layer (CLT film). Hence, it is hypothesised that the preparation of CLT/TaN coating via a combination of the PVD and EPD methods can efficiently postponement infiltration of SBF and subsequently Cl^–^ via deposited film, and hence strengthen the barrier performance. However, there are few studies concerning the effect of CLT/TaN coating on the anticorrosion property and biological activity of the Mg bare alloys. Hence, in the present research, CLT/TaN coatings were prepared on Mg alloy using PVD followed by EDP methods to enhance the cytocompatibility and anticorrosion property of the substrate in order to develop orthopedic applications of Mg alloys.

## 2. Materials and Experiments

In this research, as cast Mg-1wt% Ca-3wt%Zn alloy as the substrates with dimensions of 15 mm × 10 mm × 10 mm were fabricated according to [12], and subsequently the fabricated specimens were ground with various SiC grit papers till eventually all observable scratches were eliminated based on [12]. The TaN film was deposited by magnetron sputtering (HVC Penta Vacuum) utilizing argon gas as the sputtering gas, Ta as the sputtering target (99.95% purity), and nitrogen as the reactive gas (99.99%). The base and working pressures were 6.5 × 10^−4^ and 0.50 Pa, respectively. The overall flow rate during sputtering was 12 sccm and the power was 100 W and the preparation was carried out for 3 h without substrate heating.

**Figure 1:**
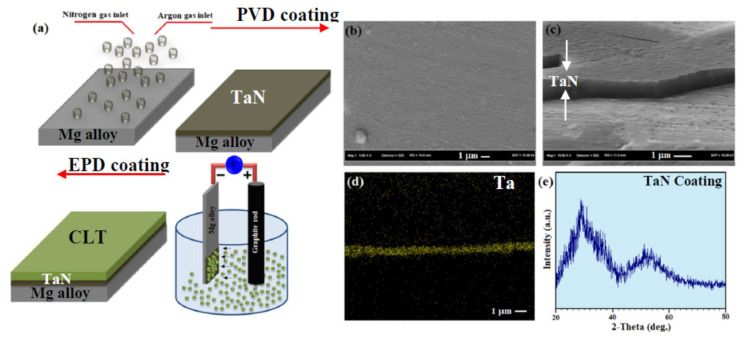

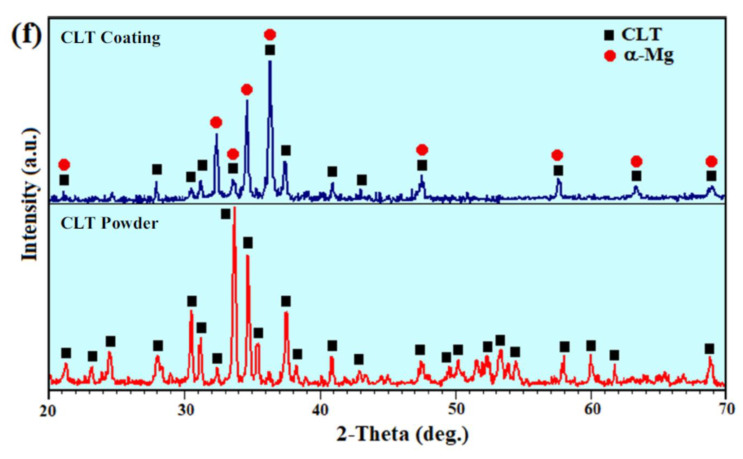
(**a**) Schematic demonstration of TaN-CLT-coated Mg alloy using PVD followed by EDP methods, (**b**) TaN coating, (**c**) cross sectional FESEM image of TaN coating, (**d**) EDS map of Ta and XRD patterns of (**e**) TaN coating and (**f**) CLT powder and CLT coating.

The CLT powders were prepared through the sol-gel method as presented in the supporting information (S1). The CLT film was deposited through the EPD procedure, associated with the standard cell along with a graphite rod and the TaN coated-Mg alloy as the anode and the cathode, respectively. The EPD solution was prepared by dissolution of CLT in methanol at an overall amount of 10 g solid/100 mL at ambient temperature under the applied voltage and electrode distance of 100 V and 15 mm, respectively. The pH of the solution was adjusted between 4.50 and 5.0 by adding HNO_3_ at ambient temperature, while the electrolyte conductivity is 1.1 μS/cm. A schematic presentation of this method is shown in Figure 1a. Scanning electron microscopy (SEM, JSM-6380LA JEOL, Tokyo, Japan) and transmission electron microscopy (TEM, HT7700 Hitachi, Tokyo, Japan) were employed to study the uncoated and CLT/TaN film microstructures. microscopy (Quanta 200 SEM, FEI Co., Eindhoven, The Netherlands). An X-ray diffractometer (D5000, Siemens, Berlin, Germany) was employed to reveal the phase components with Cu-Kα radiation (45 kV, 40 mA) over the diffraction angles (2θ) of 30–80° at a scanning speed of 4°/min. In order to determine the crystallite size of CLT powder, the Williamson–Hall equation was employed. The surface hydrophobicity of the specimens was assessed utilizing static contact angles of a water droplet placed on the surface of specimens and subsequently contact angle was rerecorded via video-based optical (VCA Optima, AST Products Inc., Billerica, MA, USA) at the room temperature with a water droplet of about 1 mL. The bonding strength of the TaN and CLT coatings was assessed based on the ASTM F1044 standard using universal testing machine (Instron 5569, Norwood, MA, USA). The electrochemical studies of the bare and coated specimens were performed using a PARSTAT 2273 potentiostat/galvanostat (Princeton Applied Research, Oak Ridge, TN, USA). The 1 cm^2^ surface area specimens were plunged in a 3-electrode cell composing of Kokubo solution (SBF) in 37 °C with a pH-value of 7.4 based on [24]. For examining the bioactivity behavior, all bare and coated samples were plunged in the cup containing 100 mm of SBF solution under 36.5 ± 1 °C conditions for 7 days. Then the specimens were brought out from the cup, and were dried in ambient conditions after rinsing with distilled water. A weight loss test was conducted to measure the corrosion rate (CR; mm/year) using the following relationship:

CR = W/Atd(1)

where W (weight loss; g), A (sample area; cm^2^), t (exposure time; s), and d (density; g/cm^3^).

In order to evaluation of cell adhesion on the CLT/TaN film, initially for sterilizing the specimens, their surfaces were coated by alcohol and subsequently placed under ultraviolet rays for 30 min. Afterwards, an MG-63 osteoblast cell line (NCBI C-555, National Cell Bank of Iran, Pasteur Institute, Tehran, Iran) at a concentration of 2 × 10^4^ cells/mL was seeded on sterilized uncoated and CLT/TaN film and cultured for 3 days and the medium was replaced by extracts with equal volume, after which the cell-scaffold constructs were stained with DAPI and afterwards presented to fluorescence image analysis. The cell toxicity of the samples was evaluated utilizing the MTT technique (an indirect method with extracts of 100% concentration) as per a previous study [25]. Three measurements were recorded and the average value was reported for each sample.

## 3. Results and Discussion

The SEM image of the Mg-Ca-Zn alloy showed that the substrate consisted of the α-Mg matrix and second phases (Mg_2_Ca) within the grain boundaries in the form of eutectic phase (α-Mg +Mg_2_Ca+Ca_2_Mg_6_Zn_3_) as can be observed in Figure S1. The surface morphology of the TaN film displayed a homogeneous and compact layer formed on the substrate surface (Figure 1b). However, a few defects including cracks are observed which could be related to low substrate temperature throughout film preparation. SEM image of cross-sectional of the TaN-coated Mg-Ca-Zn alloy sample presented formation of a uniform TaN film with strong adhesion on the alloy substrate (Figure 1c). The corresponding EDS elemental maps of film further confirmed the existence of Ta on the surface of the Mg alloy (Figure 1d). GLXRD spectrum of the deposited film exhibited the obvious peak at about 32° related to the (110) plane of TaN (JCPDS No. 71-0253) [18], while the broad peak at around of 42°–54° is assigned to the oxidized Ta with an amorphous structure (Figure 1e).

The XRD pattern of synthesized powder revealed the formation of CLT (JCPDS No. 35-0610) without the presence of impurities such as protoenstatite (Figure 1f). The crystallite size of the synthesized CLT powder was 67 nm based on the Williamson–Hall method. However, the XRD pattern of the deposited film portrayed the formation of two obvious peaks α-Mg and CLT (MgSiO_3_) phases.

**Figure 2:**
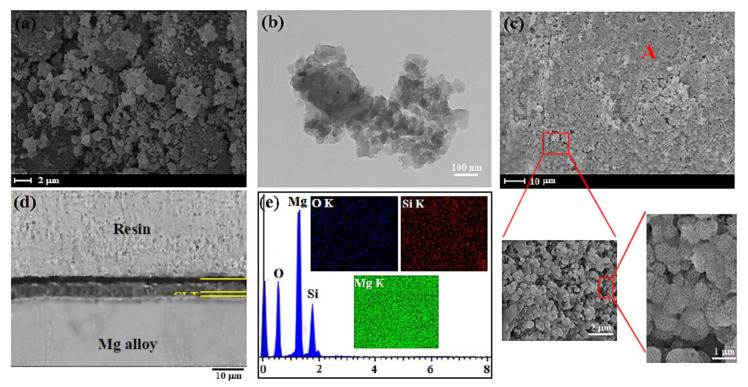
(**a**) SEM images, (**b**) TEM images of the CLT nanopowders, (**c**) CLT coated and (**d**) cross sectional FESEM image of CLT coated, and (**e**) EDS analysis of point A.

The SEM image demonstrated that the CLT powder was made up of agglomerates with homogenously distributed small particles (Figure 2a). Formation of these nanostructured particles might be caused by cold welding along with subsequent plastic deformation of the synthesized CLT powder occurred during ball-milling under the conditions which listed in Table 1. TEM image obviously illustrated that the CLT nanoparticles possess a spherical morphology with a particle size of around 60–80 nm (Figure 2b). However, after CLT coating a smooth and homogenous layer containing nano-porosity (porous structure) is formed on the surface of Mg/TaN (Figure 2c). It is actually proposed that this structure is favorable for the cell adhesion and bone tissue growth [2].

**Figure 3:**
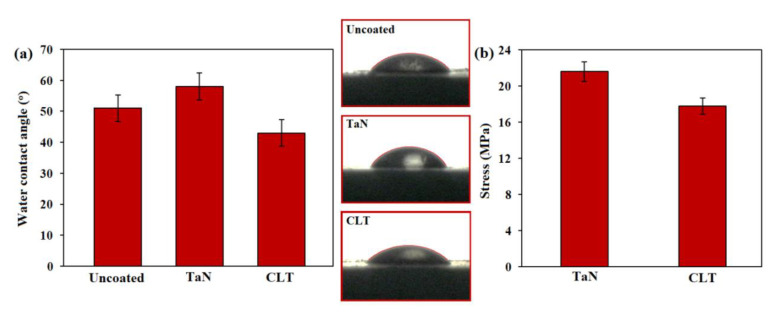
(**a**) Images of water contact angle of Mg bare alloy, TaN coated and CLT coated and (**b**) bond strengths of TaN coated and CLT coated Mg alloy.

In this respect, Tian et al. [26] have suggested that porous structures have an impact on the cellular response. In fact, this structure was regarded as an essential factor for cells growth. Similarly, Lou et al. [27] illustrated suitable porous structures could stimulate bone regeneration and enhance cell ingrowth and offer great channels for proteins and waste transportation. In the other study, Otsuki et al. [28] demonstrated that pores enable tissue and bone ingrowth, protecting against loosening, and maintaining mechanical strength of implants. The cross-sectional morphology of the fabricated CLT film showed that a smooth, homogeneous, and around 15 μm thick film was created on the TaN thin film (Figure 2d). The result of EDS analysis from prepared coating showed that the peaks of O, Si, and Mg correspond to the consisting elements of the prepared coating, implying that the CLT film was formed on the Mg alloy/TaN film (Figure 2e). Furthermore, it was noticed that the contact angle of the Mg bare alloy, TaN and CLT coatings is 51.10°, 58.30° and 43.20°, respectively, implying that the deposited film has a hydrophilic characteristic, as the water droplet effortlessly expands on the surface of specimens which it is more favorable for the cells adhesion (Figure 3a).

The adhesive strength (Figure 3b) of the TaN and CLT coated Mg alloy samples was about 21.1 and 17.8 MPa, respectively, which is required for orthopaedic implant applications (15 MPa) [1]. The greater adhesive strength of TaN is attributed to the creation of thin films which were deposited via PVD method.

**Figure 4:**
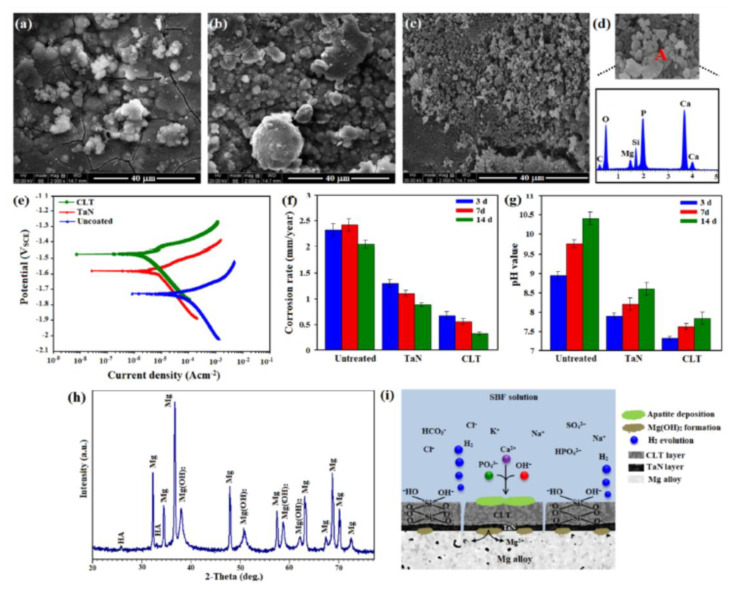
SEM micrograph of (**a**) Mg bare alloy, (**b**) TaN coated, (**c**) CLT coated after immersion into Kokubo for 14 days and (**d**) EDS analysis of A, (**e**) Potentiodynamic polarization curves, (**f**) corrosion rate, (**g**) pH value for Mg bare alloy, TaN coated and CLT coated Mg alloy and (**h**) XRD patterns and (**i**) Schematic demonstration of the corrosion mechanism of CLT coated Mg alloy after immersion in SBF solution for 14 days.

In the SEM image of Mg bare alloys, it is likely to observe that severe corrosion of the alloy has happened following incubation for long-time while mild corrosion attack was noticed on the coated surface (Figure 4a–c). From the EDS spectra of the sample surface (Figure 4d) and cross-section of CLT coated-sample (Figure S2), it is likely to be detected that the created film consisted of Ca, P, Mg, C and O, suggesting the creation of hydroxyapatite during the incubation. As can be seen in Figure 4e, the CLT deposited sample displayed diminished kinetics of anodic and cathodic reactions. Their *i*_corr_ dramatically diminished after the formation of protective film (CLT and TaN) compared to the bare magnesium (168.72 μA/cm^2^), indicating a substantial enhancement in the anticorrosion property after the CLT deposition. The CLT/TaN coating specimen exhibited a lower *i*_corr_ (3.78 μA/cm^2^) than that of the TiO_2_ coating (47.7 μA/cm^2^) [12] and the PEO coating (7.65 μA/cm^2^) [24]. Similarly, more noble corrosion potential (E_corr_) for the CLT and TaN coated samples compared with the Mg bare alloy displayed that better protection could be provided by CLT coating. The combination of CLT and TaN films would diminish the solution penetration into the substrate, causing a considerable reduction in the anticorrosion property of the bi-layer coating sample. In fact, CLT/TaN coating substantially slowdown the infiltration of SBF and subsequently postponed the incident of pitting corrosion. This may be attributed to the presence of CLT as an over-layer on the TaN as an under-layer. On the flip side, the presence of TaN as an under-layer with a smaller number of cracks, higher compactness and more homogeneous on substrate surface might protect from the substrate via decline of transportation of SBF solution containing Cl^–^ to the Mg alloy substrate.

According to Figure 4f, the corrosion rate of the Mg sample was substantially greater when compared with that of the TaN and CLT coated samples throughout the entire immersion time. This graph evidently implies that the anticorrosion property was remarkably increased after CLT deposition. In addition, the corrosion rate of all groups elevated in the short time, although decreased dramatically between 72 and 168 h which could be explained by the formation of apatite that protects the substrate for long time. Regarding TaN and CLT deposited samples, these deposited films worked as barriers which will be able to protect the substrate from the solution penetration into the substrate. Figure 4g showed the variation of the pH value throughout the incubation time of Mg bare alloy, TaN and CLT deposited samples. A similar pattern was noticed in the pH value for the entire specimens. In the early time of incubation (i.e., 3 days), all samples including bare Mg alloy, TaN coating and CLT coating are in great contact with the SBF and therefore are inclined to be compromised via the SBF, and escalation in pH is noticed. After 14 days, corrosion product was deposited on the surface of all samples which work as barrier layer, which resulted in the pH values escalation rate is reduced. When the incubation finished, the pH value of bare Mg alloy, TaN coating and CLT coating is 10.35, 8.62 and 7.91 respectively implying lower pH value of CLT coating in comparison with the bare Mg alloy [8,10,19]. The XRD pattern of the CLT coating samples after incubation in the SBF for 14 days depicted the existence of the sharp peaks of Mg(OH)_2_ as well as the small peaks of HA, along with the α-Mg peaks which is related to the matrix (Figure 4h). The existence of HA peaks implying CLT coating has great capability for formation of apatite in the SBF. This may owing to the generation of Si and Mg ions from the CLT coating which might stimulate the apatite formation. Figure 4i is a schematic illustration of the formation of bone-like apatite on the TaN/CLT deposited samples in the SBF solution. After dissolving CLT, loss of soluble silica in the form of Si(OH)_4_ in the solution, causes changes in Si–O–Si bonds and formation of Si–OH (silanol group) at the CLT coated surface as Equation (2):

Si–O–Si + H_2_O → Si–OH + HO–Si (2)

In this regard, the incorporation of Si-OH groups causes the creation of a thin layer of SiO_2_ hydrated gel, which eventually uses alkaline cations. However, the thin gel layer is negative, and it might react with PO_4_^3−^ and Ca^2+^ ions in the electrolyte [19,24]. At the same time, Mg^2+^ and Ca^2+^ ions and OH^−^ ions dissolve in the surface and constantly enhance to get through to the solubility degree of apatite in the electrolyte based on the following Equation (3):

10Ca^2+^ + 6PO_4_^3−^ + 2OH^−^ → Ca_10_ (PO_4_)_6_(OH)_2_ (3)

**Figure 5:**
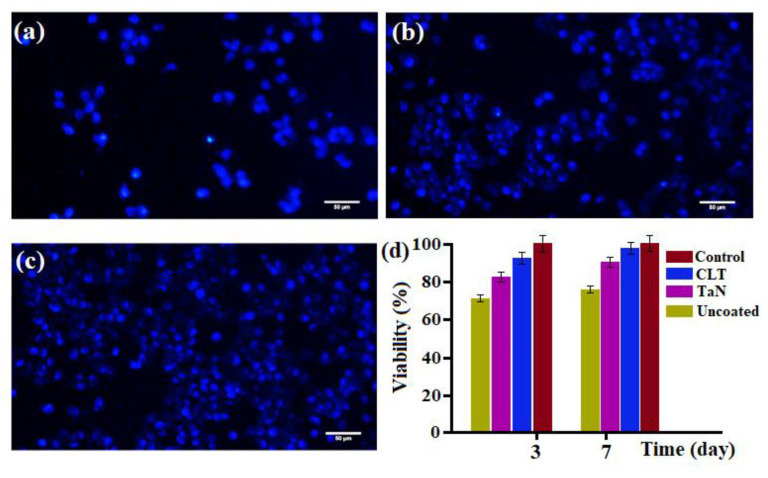
DAPI staining of MG63 osteoblasts cells on (**a**) Mg bare alloy, (**b**) TaN coated, (**c**) CLT coated, and (**d**) Cell viability of MG63 osteoblast cells after incubation in uncoated and coated Mg alloy specimens for 3 and 7 days.

The fluorescence images of MG63 cultured on the Mg bare alloy and TaN and CLT deposited samples after 3 days are shown in Figure 5a–c. 

It was found that there were greater number of cells (stained blue) on TaN and CLT deposited samples compared to the Mg bare alloy. This implies the great biocompatibility of the TaN/CLT deposited samples, whereas the cells cultured on the bare sample failed to adhere and spread well because of its higher dissolution rate, formation of a great amount of corrosion product and high pH value when exposed to the solution [29,30,31,32]. For better assessment of the biocompatibility of the specimens, an MTT assay was additionally conducted. Cellular viability was assessed using the MTT assay and the result revealed that TaN and CLT coated extract have less significant impact on cell viability, whereas exposing the bare Mg alloy extract into the medium generated marginally cytotoxicity of the cells. Figure 5d clearly showed greater cell viability of TaN and CLT deposited samples in comparison with the uncoated sample. Among the coating groups, the CLT deposited sample presented the highest Mg-63 cell viability (above 90%) owing to the dissolution of CLT ionic products containing Mg and Si ions into the physiological medium, will cause greater cell attachment and growth.

## 4. Conclusions

Effective combination methods composed of PVD and EPD was employed to deposit homogeneous and uniform TaN and CLT with a thickness around 15 μm on the surface of Mg-Ca-Zn alloy with the aim of escalating the anticorrosion property and biocompatibility. The nano thin TaN under-layer (950 nm) depicted a more compact structure with less porosity and cracks compared to the thick CLT top-layer. Deposition of CLT/TaN film on the Mg-Ca-Zn alloy surface significantly escalates E_corr_ and reduces *i*_corr_ of the substrate, implying substantial enhancement in the corrosion characteristic. A model was suggested to reveal the degradation process of the CLT/TaN coated-layer. The DAPI staining exhibited higher osteoblast cells density on CLT/TaN and TaN coatings than the Mg-Ca-Zn bare alloy. This study showed combination of PVD and EPD as an effective surface treatment method for Mg-based alloys and provide feasible mechanisms of the Mg alloy-coated degradation. 

## Figures and Tables

**Table 1:** Mechanical alloying parameters adopted for milling CLT powder.

Parameter	Value
Rotation speed	300 rpm
Ball-powder weight ratio	10:1
Mass of powder	10 g
Milling time	10 h
Capacity of vial	125 mL
Diameter of the balls	10 mm and 20 mm

## Supplementary Materials

The following are available online at https://www.mdpi.com/2079-4983/11/2/26/s1, Figure S1: (a) SEM images of as-cast Mg-1Ca-3Zn alloy, (b) EDS analysis of Mg-1Ca-3Zn alloys (Spectrum 2) and (c) X-ray diffraction pattern of Mg-1Ca-3Zn alloys, Figure S2: EDS spectrum of the cross-section of CLT coated-sample after immersion into SBF for 14 days.
