# Supplementary material for: Clinoenstatite/Tantalum Coating for Enhancement of Biocompatibility and Corrosion Protection of Mg Alloy
Source: J Funct Biomater. 2020 Apr 13;11(2):26. doi: 10.3390/jfb11020026 (PMC7353525; doi:10.3390/jfb11020026)
Supplement: Supplementary file 1 [file jfb-11-00026-s001.pdf]

Supporting Information

S1 Clinoenstatite preparation: Clinoenstatite powders were prepared by precipitation process using magnesium nitrate ($Mg(NO_3)_2·6H_2O$) and sodium silicate ($Na_2SiO_3·9H_2O$) as raw materials (molar ratio: $Mg(NO_3)_2·6H_2O/Na_2SiO_3·9H_2O$ = 1:1). Each raw material was dissolved in water with a concentration of 0.5 mol/L. Magnesium nitrate solution was added into sodium silicate solution dropwise. After the precipitation process, the suspension was filtered and washed with distilled water and ethanol, and dried at 60 °C for 2 days to obtain the dry powders. The dry powders were sieved through a 250 mesh screen, and calcined at 1300 °C. In order to fabricate nanostructured CLT, the powder was milled in a planetary ball mill (Retsch PM400) under the conditions which listed in Table 1.

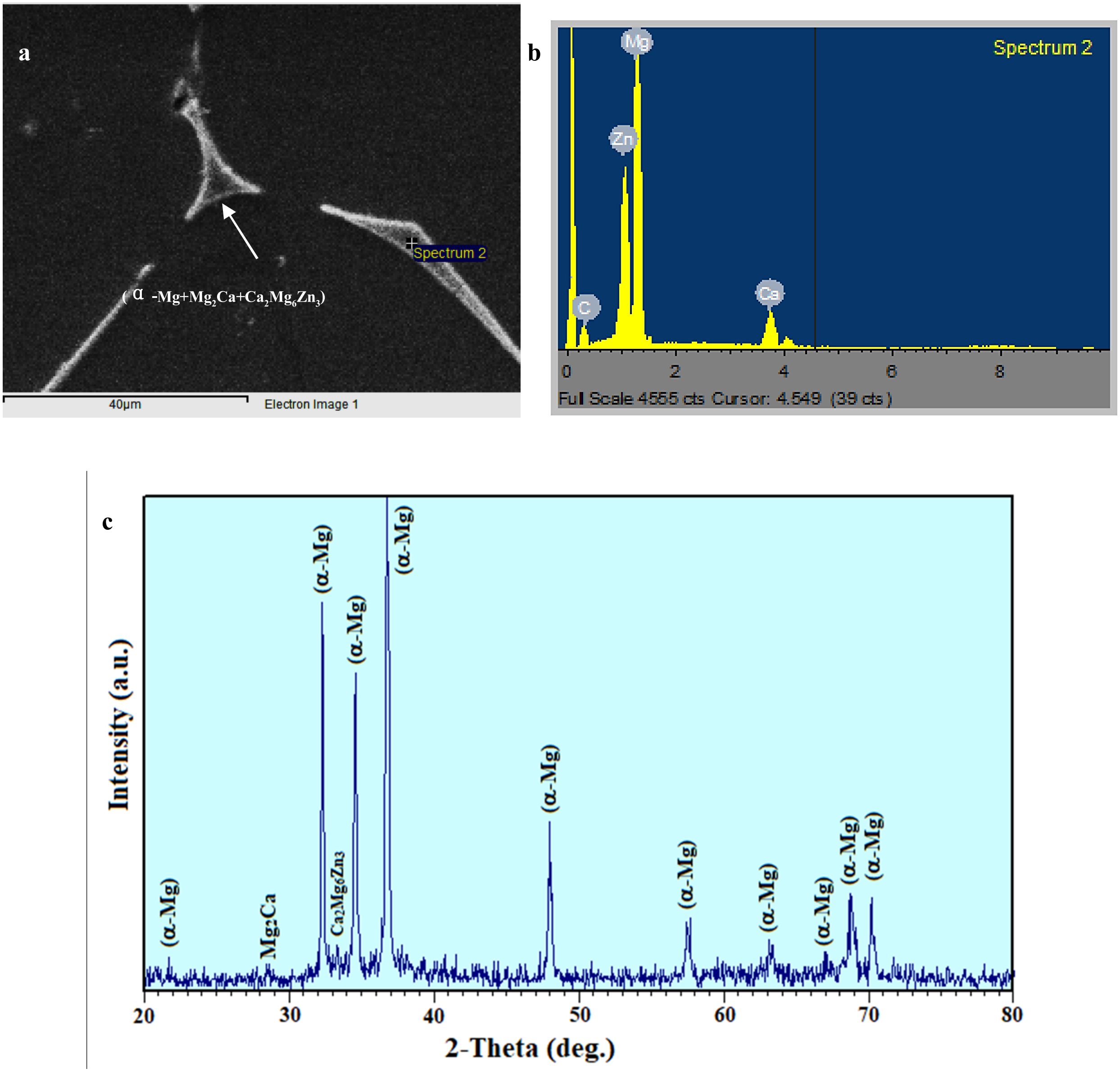

Figure S1. (a) SEM images of as-cast Mg-1Ca-3Zn alloy, (b) EDS analysis of Mg-1Ca-3Zn alloys (Spectrum 2) and (c) X-ray diffraction pattern of Mg-1Ca-3Zn alloys.

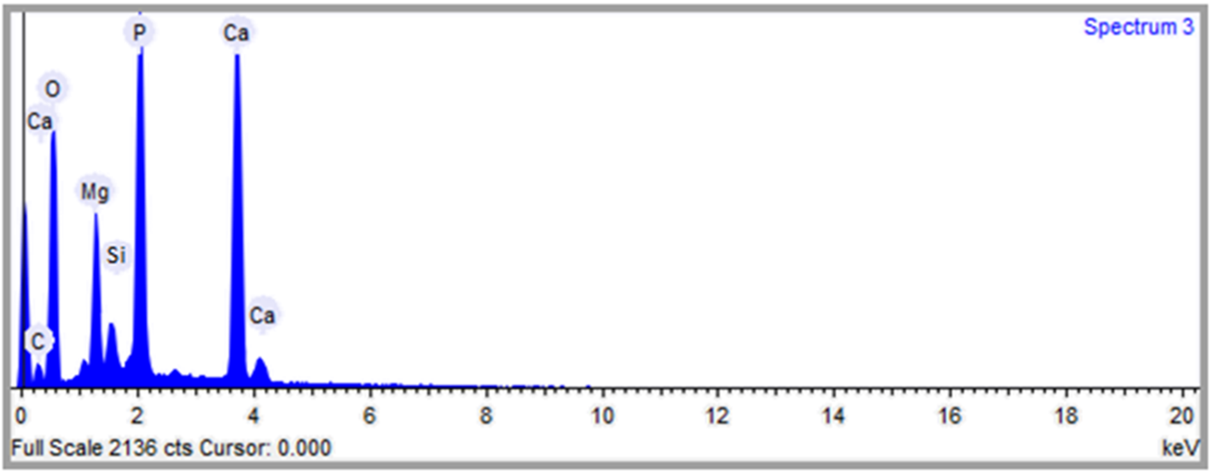

Figure S2. EDS spectrum of the cross-section of CLT coated-sample after immersion into SBF for 14 days.
